# Situs Inversus Totalis and Severe Early‐Onset Developmental Epileptic Encephalopathy in a Child With a Homozygous CFAP52 Mutation: A Case Report

**DOI:** 10.1002/ccr3.73165

**Published:** 2026-07-19

**Authors:** Anwar Abu Hetta, Lina Abughaboosh, Jenan Al‐Qasrawi, Balqees Amro, Mayar Alshaer

**Affiliations:** ^1^ Department of Pediatrics Hebron Governmental Hospital Hebron Palestine; ^2^ College of Medicine and Health Sciences Palestine Polytechnic University Hebron Palestine

**Keywords:** CFAP52, ciliopathy, developmental epileptic encephalopathy, genetic mutation, heterotaxy type 10, situs inversus totalis

## Abstract

We report a 2‐year‐old boy with a homozygous CFAP52 mutation presenting with situs inversus totalis, severe epileptic developmental encephalopathy, and global developmental delay, expanding the phenotypic spectrum of CFAP52‐related ciliopathies. Clinical findings included multifocal epileptiform discharges, thin corpus callosum, and a small subdural hematoma. Trio whole‐exome sequencing also revealed a homozygous NCAPG2 variant of uncertain significance, however, its clinical significance remains unproven and no causal relationship can be established. This case highlights the diagnostic value of comprehensive genomic testing in complex neuro‐visceral disorders and underscores the importance of cautious interpretation of co‐inherited variants in atypical ciliopathy presentations. The report follows CARE guidelines.

## Key Clinical Message

Children presenting with situs inversus and severe early‐onset seizures warrant prompt neurological assessment and comprehensive genomic testing. This case illustrates how trio whole‐exome sequencing can clarify complex neuro‐visceral phenotypes and guide clinical management beyond classical gene–phenotype associations.

## Graphical abstract

Serial sagittal T1‐weighted MR images demonstrating persistent thinning of the corpus callosum. (A) Initial MRI at 19 months of age shows diffuse thinning of the corpus callosum involving the body and splenium. (B) Follow‐up MRI one year later demonstrates stable appearance without interval progression, consistent with corpus callosal hypoplasia.

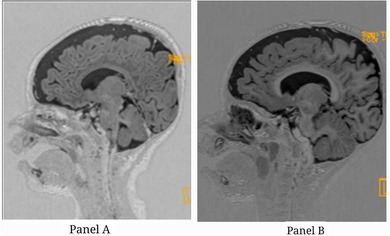

## 1 Introduction

Ciliopathies are a broad category of conditions resulting from irregularities in the structure or function of both motile and non‐motile cilia. These organelles are essential for the development of the embryo, especially for the establishment of left–right body asymmetry [1, 2]. Multiple organ involvement and visceral heterotaxy can result from mutations in ciliary genes, including CFAP52 [3, 4].

Heterotaxy 10 results from a mutation in the CFAP52 gene, which codes for a protein necessary for the motility and structural integrity of cilia and flagella [4, 5]. An unusual association between global developmental delay and severe neurological symptoms, such as developmental and epileptic encephalopathy, raises the possibility of broader neurodevelopmental involvement, although a direct causal role of CFAP52 in this phenotype has not been established.

## 2 Case History and Examination

A 2‐year‐and‐5‐month‐old boy presented with recurrent generalized tonic–clonic seizures of two‐minute duration, beginning 3 days prior to admission.

He was born at full term via vacuum‐assisted vaginal delivery, with a birth weight of 3750 g. The neonatal course was notable for tachypnea requiring a 10‐day NICU stay. The parents were non‐consanguineous; this was their first child, and there was no family history of neurodevelopmental disorder.

Prenatal imaging revealed situs inversus totalis with dextrocardia. Postnatal echocardiography demonstrated a small patent foramen ovale (PFO) and patent ductus arteriosus (PDA). There was no significant family history of neurological disease.

Developmentally, the child exhibited global developmental delay since infancy, with delayed motor and language milestones.

On physical examination, he was well nourished with no dysmorphic features. Cardiovascular examination confirmed dextrocardia. Neurological examination revealed global developmental delay without focal neurological deficits.

## 3 Differential Diagnosis, Investigations, and Treatment

### 3.1 Differential Diagnosis

The differential diagnoses included:

- Primary ciliary dyskinesia
- Joubert syndrome
- Other syndromic ciliopathies associated with neurodevelopmental delay
- Genetic epileptic encephalopathies

### 3.2 Investigations

Initial laboratory investigations, including serum calcium (10.2 mg/dL), magnesium (2.1 mg/dL), sodium (134 mmol/L), potassium (4.9 mmol/L), chloride (104 mmol/L), lactate (24), ammonia (19.2), and coagulation profile, were within normal limits.

Initial electroencephalography (EEG) demonstrated multifocal epileptiform discharges, predominantly over the right hemisphere, with a burst‐suppression pattern, consistent with epileptic encephalopathy. A representative EEG tracing is shown in Figure 1. Because seizures persisted despite initial antiseizure therapy, a follow‐up EEG was performed and demonstrated findings consistent with West syndrome, prompting modification of the antiseizure treatment regimen.

**FIGURE 1:**
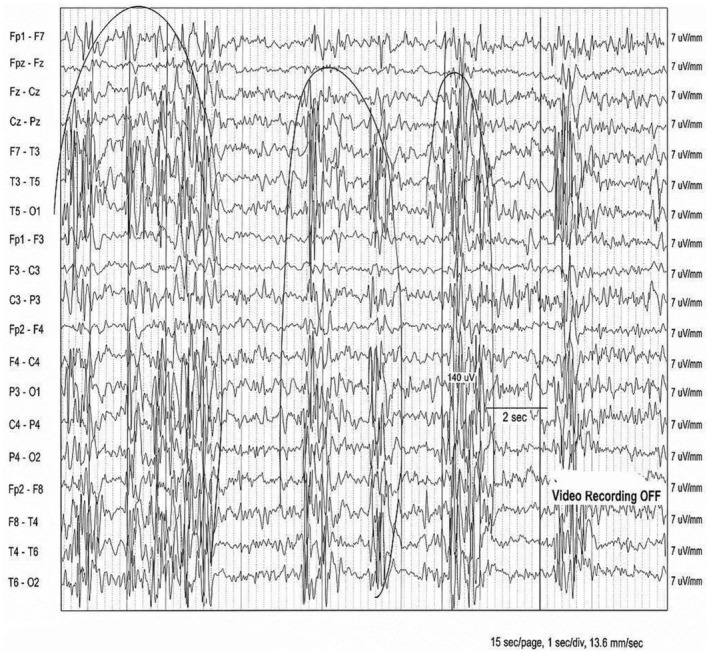
Representative EEG tracing showing markedly abnormal background activity with multifocal epileptiform discharges, more prominent over the right hemisphere. Calibration is shown on the tracing (140 μV, 2 s).

Brain MRI revealed a right fronto‐parietal subdural hematoma measuring 0.8 cm in thickness and 5.5 cm in length, with mild compression of the right lateral ventricle (Figure 2). Prominent subarachnoid spaces were observed, consistent with benign enlargement of the subarachnoid spaces (BESS). The corpus callosum appeared thin, measuring approximately 2.8 mm at the genu.

**FIGURE 2:**
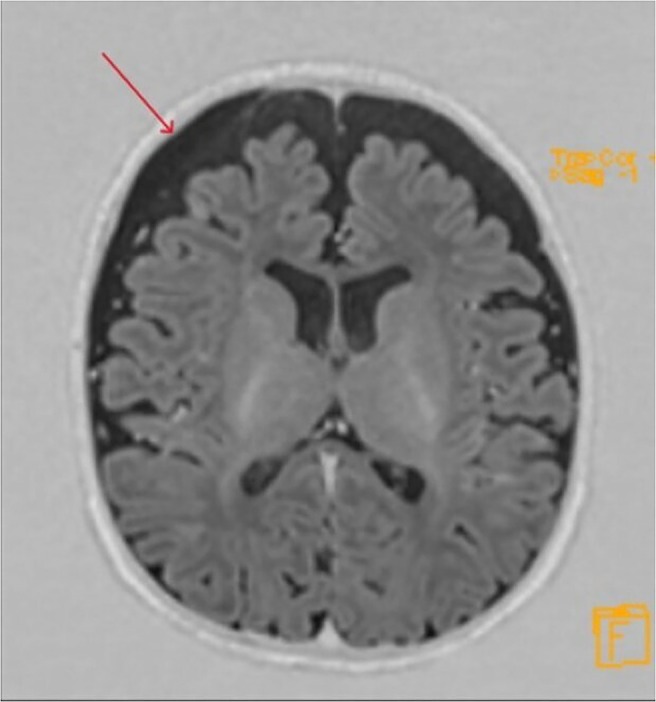
Axial T1‐weighted MRI showing a thin right subdural hematoma (arrow) with mild compression of the right lateral ventricle. Note the prominent subarachnoid spaces over both frontal convexities, consistent with benign enlargement of the subarachnoid spaces (BESS).

Serial sagittal T1‐weighted MRI confirmed persistent thinning of the corpus callosum without interval progression (Figure 3).

**FIGURE 3:**
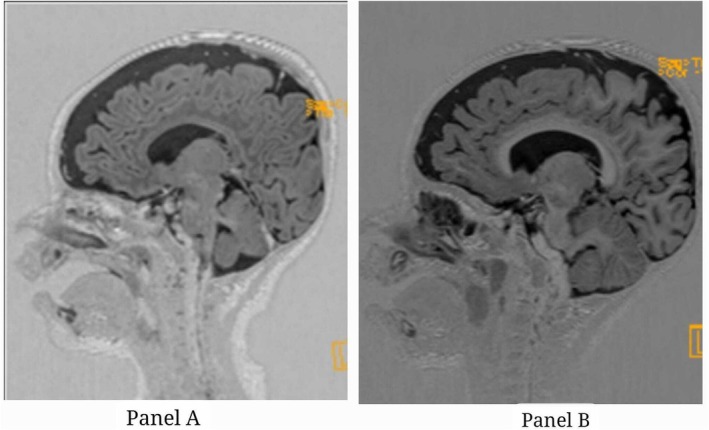
Serial sagittal T1‐weighted MR images demonstrating persistent thinning of the corpus callosum. (A) Initial MRI at 19 months of age shows diffuse thinning of the corpus callosum involving the body and splenium. (B) Follow‐up MRI 1 year later demonstrates stable appearance without interval progression, consistent with corpus callosal hypoplasia.

Given the complex neuro‐visceral phenotype and limited local neurogenetic resources, trio whole‐exome sequencing (WES) was performed. Two findings were identified:

1. A homozygous pathogenic CFAP52 missense variant (c.811G>A; p.Gly271Arg; NM_145054.5) was identified in the proband. Segregation analysis showed that both parents were heterozygous carriers, consistent with autosomal recessive inheritance.
2. A homozygous variant of uncertain significance in NCAPG2 (NM_017760.7:c.2944T>C; p.Ser982Pro) was also identified. Although NCAPG2 is associated with Khan–Katsanis syndrome, the clinical significance of this specific variant remains uncertain.

### 3.3 Treatment

Acute seizure management was initiated with intravenous phenobarbital (20 mg/kg loading dose), followed by maintenance therapy (15 mg every morning and 22.5 mg every evening). Vitamin B6 (100 mg daily) was also administered while potentially treatable causes of infantile epilepsy were being evaluated.

Because seizures persisted despite phenobarbital therapy, levetiracetam (150 mg every 12 h) and clobazam (0.25 mg once daily) were added.

At baseline, the patient experienced approximately 4–6 seizure episodes per day despite initial therapy.

Following repeat EEG findings suggestive of West syndrome, phenobarbital was discontinued and vigabatrin was initiated, with the dose titrated from 250 mg once daily to 250 mg twice daily, in accordance with the suspected electroclinical diagnosis.

After optimization of the antiepileptic regimen, seizure frequency decreased to approximately 1–2 brief seizure episodes per week without achieving seizure freedom.

The patient experienced mild sedation during treatment.

No formal epilepsy gene panel was performed; instead, genetic evaluation proceeded directly with trio whole‐exome sequencing due to the complex neuro‐visceral presentation. Comprehensive multidisciplinary rehabilitation, including physiotherapy, developmental intervention, cognitive stimulation, and feeding support, was recommended.

## 4 Conclusion and Results (Outcome and Follow‐Up)

During follow‐up, seizures showed partial clinical improvement under combination antiepileptic therapy. Developmental delay remained evident. The patient was able to sit with support but remained unable to roll over independently or reach for objects appropriately for age. Eye contact was preserved, and he responded to auditory stimuli by turning toward sounds; however, social interaction and developmental progress remained markedly delayed.

Follow‐up neuroimaging demonstrated no progression of the chronic subdural collection, and thinning of the corpus callosum remained stable without additional structural abnormalities.

## 5 Discussion

Ciliopathies encompass a heterogeneous group of genetic disorders caused by dysfunction of motile or primary cilia, resulting in diverse systemic and neurological phenotypes [1, 2]. The CFAP52 gene (also known as WDR16) encodes a WD‐repeat–containing protein localized to basal bodies and centrosomes, structures essential for proper ciliary assembly and motility [6, 7].

Mutations in CFAP52 have been previously associated with laterality disorders. Ta‐Shma et al. reported homozygous deletions in WDR16 in patients with complex situs anomalies, confirming its role in left–right asymmetry [8]. Similarly, Postema et al. identified CFAP52 variants among rare causes of situs inversus totalis without primary ciliary dyskinesia [9].

The current case extends the CFAP52‐associated phenotype beyond laterality defects, describing severe epileptic encephalopathy and neurodevelopmental delay. Tapia Contreras et al. showed that CFAP52/WDR16 localizes to basal bodies and is required for spermatogenesis and flagellar formation [6], while Jin et al. demonstrated that CFAP52 mutations disrupt flagellar and cellular architecture [7]. These data suggest that CFAP52 dysfunction may impair not only visceral patterning but also neuronal organization.

Notably, our patient also harbored a homozygous NCAPG2 missense variant (c.2944T>C; p.Ser982Pro), which was classified as a variant of uncertain significance (VUS). NCAPG2 encodes a core subunit of the condensin II complex and is essential for chromosome condensation and faithful chromosome segregation during mitosis. Pathogenic biallelic variants in NCAPG2 have been associated with Khan–Katsanis syndrome (3KS), a rare autosomal recessive neurodevelopmental disorder characterized by severe developmental impairment, microcephaly, ocular abnormalities, and congenital anomalies [10]. However, the p.Ser982Pro variant has not been established as disease‐causing. Franklin classification data support a VUS interpretation, with extremely low population frequency in gnomAD (maximum reported frequency 0.0%, fulfilling PM2), but without computational evidence sufficient to support either pathogenicity or benignity (aggregated in silico prediction score 0.324; PP3 and BP4 unmet) [11]. In addition, parental segregation analysis for NCAPG2 was not performed. Therefore, the available evidence supports cautious interpretation of this finding as a co‐inherited VUS rather than a demonstrated modifier or second molecular diagnosis, and its contribution to the patient's neurodevelopmental phenotype cannot be determined at present.

Brain MRI demonstrated a right chronic subdural hematoma in association with benign enlargement of the subarachnoid spaces (BESS). In infants, BESS is a recognized predisposing factor for subdural collections, likely related to stretching of the bridging veins, and the presence of a subdural hematoma in this setting is not pathognomonic for non‐accidental trauma [12, 13]. In our patient, coagulation studies were normal, and no bleeding diathesis was identified. Therefore, the exact etiology of the subdural collection remains uncertain. Although the history of vacuum‐assisted delivery may suggest a possible perinatal traumatic contribution, the imaging findings are also compatible with BESS as a predisposing factor. Accordingly, the subdural hematoma should be interpreted as a potentially contributory but not definitive finding, and a direct causal relationship with the underlying genetic diagnosis cannot be established [12, 13, 14].

Motile cilia are crucial not only for establishing visceral laterality but also for neuronal organization and brain development. During embryogenesis, nodal motile cilia generate a leftward fluid flow that determines left–right body asymmetry, including organ situs. In the central nervous system, ependymal cilia lining the ventricular system are essential for maintaining cerebrospinal fluid (CSF) circulation and for influencing neuronal migration and cortical patterning [15, 16, 17, 18]. Disruption of these ciliary functions can impair neurodevelopmental processes, potentially leading to cortical dysgenesis and epileptogenesis, as observed in the present case.

To our knowledge, this is the first reported case linking a homozygous CFAP52 mutation with severe early‐onset epileptic encephalopathy, thereby expanding the neurological phenotype of CFAP52‐related ciliopathies. This report reinforces the diagnostic importance of comprehensive genomic testing—especially trio exome sequencing—in children presenting with combined systemic and neurological manifestations. Such approaches facilitate precision diagnosis, family counseling, and the identification of emerging gene–phenotype correlations.

### 5.1 Strengths and Limitations

This report highlights the identification of a homozygous CFAP52 variant in a patient with severe early‐onset developmental and epileptic encephalopathy, together with a co‐inherited NCAPG2 variant of uncertain significance. Its strengths include the detailed clinico‐radiologic‐genetic correlation and the documentation of an expanded phenotype. Its limitations include the single‐case design, the lack of functional studies, and the absence of electron microscopy or other ultrastructural confirmation of ciliary abnormalities. In addition, the contribution of the NCAPG2 variant remains uncertain, and a modifier effect has not been demonstrated.

### 5.2 Clinical Relevance

This case broadens the reported clinical spectrum of CFAP52‐related ciliopathies while underscoring that the association with NCAPG2 remains unproven. It highlights the value of comprehensive genomic testing, including trio exome sequencing, in children with complex neuro‐visceral phenotypes and the need for cautious interpretation of variants of uncertain significance in clinical decision‐making.

The report conforms to the CARE guidelines for case reporting [19].

## 6 Conclusion

This case expands the clinical spectrum of CFAP52‐related ciliopathies by suggesting that the phenotype may include severe neurodevelopmental involvement in addition to laterality defects. The coexisting NCAPG2 variant remains a variant of uncertain significance, and its contribution to the phenotype cannot be determined from the available evidence. This report highlights the value of trio whole‐exome sequencing in children with complex neuro‐visceral presentations and underscores the need for cautious interpretation of co‐inherited variants in the absence of functional validation.

## Author Contributions

**Mayar Alshaer:** data curation, investigation, writing – original draft. **Lina Abughaboosh:** conceptualization, methodology, project administration, supervision, validation, writing – original draft, writing – review and editing. **Anwar Abu Hetta:** conceptualization, data curation, supervision, writing – review and editing. **Balqees Amro:** data curation, investigation, writing – original draft. **Jenan Al‐Qasrawi:** data curation, investigation, writing – original draft.

## Funding

The authors have nothing to report.

## Ethics Statement

Written informed consent for publication was obtained from the patient's legal guardians.

## Consent

Written informed consent for publication was obtained from the patient's legal guardians.

## Conflicts of Interest

The authors declare no conflicts of interest.

## Data Availability

All relevant clinical and imaging data supporting the findings of this case report are included within the article. Genetic data are available from the corresponding author upon reasonable request, in accordance with ethical and privacy regulations.
